# The structure of inactivated mature tick-borne encephalitis virus at 3.0 Å resolution

**DOI:** 10.1080/22221751.2024.2313849

**Published:** 2024-03-11

**Authors:** Evgeny B. Pichkur, Mikhail F. Vorovitch, Alla L. Ivanova, Elena V. Protopopova, Valery B. Loktev, Dmitry I. Osolodkin, Aydar A. Ishmukhametov, Valeriya R. Samygina

**Affiliations:** a NRC «Kurchatov Institute», Moscow, Russian Federation; b FSASI “Chumakov FSC R&D IBP RAS” (Institute of Poliomyelitis), Moscow, Russian Federation; c Institute of Translational Medicine and Biotechnology, Sechenov First Moscow State Medical University, Moscow, Russian Federation; d State Research Center of Virology and Biotechnology “Vector”, Novosibirsk, Russian Federation

**Keywords:** Cryo-electron microscopy, inactivated vaccines, structural virology, tick-borne encephalitis virus

## Abstract

Tick-borne encephalitis virus (TBEV) causes a severe disease, tick-borne encephalitis (TBE), that has a substantial epidemiological importance for Northern Eurasia. Between 10,000 and 15,000 TBE cases are registered annually despite the availability of effective formaldehyde-inactivated full-virion vaccines due to insufficient vaccination coverage, as well as sporadic cases of vaccine breakthrough. The development of improved vaccines would benefit from the atomic resolution structure of the antigen. Here we report the refined single-particle cryo-electron microscopy (cryo-EM) structure of the inactivated mature TBEV vaccine strain Sofjin–Chumakov (Far-Eastern subtype) at a resolution of 3.0 Å. The increase of the resolution with respect to the previously published structures of TBEV strains Hypr and Kuutsalo-14 (European subtype) was reached due to improvement of the virus sample quality achieved by the optimized preparation methods. All the surface epitopes of TBEV were structurally conserved in the inactivated virions. ELISA studies with monoclonal antibodies supported the hypothesis of TBEV protein shell cross-linking upon inactivation with formaldehyde.

## Introduction

Tick-borne encephalitis virus (*Orthoflavivirus encephalitidis*, TBEV) is an enveloped single-stranded (+)RNA virus belonging to the *Orthoflavivirus* genus [1,2]. Seven TBEV subtypes were distinguished based on the 10% nucleotide sequence diversity threshold, and three of them are most prominent and widely distributed: European (TBEV-Eur), Siberian (TBEV-Sib), and Far-Eastern (TBEV-FE), named according to their major geographic distribution regions [3]. After transmission into a human via an infected tick bite or other means, TBEV infection may cause tick-borne encephalitis (TBE), which has a potential to cause a long-term disability, negatively affecting the patient’s quality of life [1]. TBE manifestations vary from uncomplicated fevers to potentially lethal encephalitis or meningoencephalitis coupled with paralysis; TBEV-Eur infections are usually mild, while TBEV-Sib and TBEV-FE cause more severe disease [1,4]. A chronic TBE form manifests with lifelong neurological symptoms [1].

Despite a limited number of diagnosed cases (thousands yearly), TBE prevention and treatment are important problems of the current medicine. Several inactivated TBE vaccines based on TBEV-Eur or TBEV-FE strains are widely used in the clinical practice, showing a good safety and efficacy profile [1,4,5], although cases of vaccine breakthrough occur sporadically [4–9]. Animal studies showed that these cases may be caused by both host-related and virus-related factors, such as immune status of the host or the individual properties of the vaccine and challenge virus strains [10,11]. Numerous small molecule compounds were assessed as potential direct-acting antivirals targeting TBEV [12], although their clinical applicability has not yet been studied. Thus the development of improved TBE vaccines and antiviral drugs is essential. The combination of a high-resolution 3D structure with functional analysis of the structural proteins forming the viral particle would establish a solid basis for the rational design of advanced TBE vaccine antigens and antivirals.

Orthoflavivirus particles have icosahedral symmetry with a diameter of about 50 nm and a genome of about 11 kb. The genome is packaged into a nucleocapsid enclosed within a host-derived lipid membrane and encodes three structural proteins: (1) envelope (E), (2) pre-membrane (prM), and (3) capsid (C), as well as seven nonstructural proteins [13]. The glycoprotein shell of the mature infectious virion consists of 180 copies of each of membrane-anchored E (496 amino acid residues) and M proteins (75 amino acid residues). Immature orthoflavivirus particles have a spiky appearance with 60 trimers of E:prM heterodimers exposed [14,15]. prM protein functions as a chaperone during virion maturation, which is achieved in the trans-Golgi compartment and finalized after the cleavage of prM by the host protease furin to remove the pr peptide in the extracellular environment. The mature particles are infectious and may enter the cells via endocytosis [16–18]. Envelope proteins E orchestrate the early stages of viral infection, virus entry into the cell and receptor-mediated endocytosis [19–21]. E proteins undergo dramatical pH-induced conformational changes in the low pH environment of endosomes, where the smooth mature particles with 90 dimers of E:M heterodimers on the surface reorganize to expose the fusion peptides that attack the membrane of the endosome to initiate the membrane fusion [22] followed by the virus genome release into the cytoplasm.

Previously, the following TBEV structures were solved with the help of cryo-electron microscopy (cryo-EM) approach: a 3.9 Å structure of intact TBEV strain Hypr (PDB ID 5O6A [23], TBEV-Hypr), a 3.9 Å structure of TBEV strain Hypr complexed with Fab fragment of neutralizing antibody 19/1786 (PDB ID 5O6V [23]), a 3.3 Å structure of UV-inactivated TBEV strain Kuutsalo-14 (PDB ID 7Z51 [24], TBEV-Kuutsalo). Structures of immature TBEV virions from strains Kuutsalo-14, Neudoerfl, and Hypr (resolution 4–8 Å) have been described but not formally published yet [25]. All of these structures represent the European subtype of TBEV. In this study, we performed the first cryo-EM structure determination for the particles from the clinically approved TBE vaccine preparation based on a Far-Eastern subtype strain. It demonstrated the reliability of virus samples inactivated by cross-linking agents for structure determination and surface epitope analysis, which is especially important for emerging viruses. We have also compared the accessibility of epitopes for intact and inactivated TBEV by means of enzyme-linked immunosorbent assay (ELISA) to corroborate the structural findings.

## Results

### Virion structure

Formaldehyde-inactivated TBEV strain Sofjin-Chumakov is produced in the industrial scale for the vaccine manufacturing process and represents its antigen. We prepared a purified and concentrated iTBEV suspension (Supplementary Figure S1) according to the previously optimized protocol [26] and used it for the structure determination.

**Figure 1.**
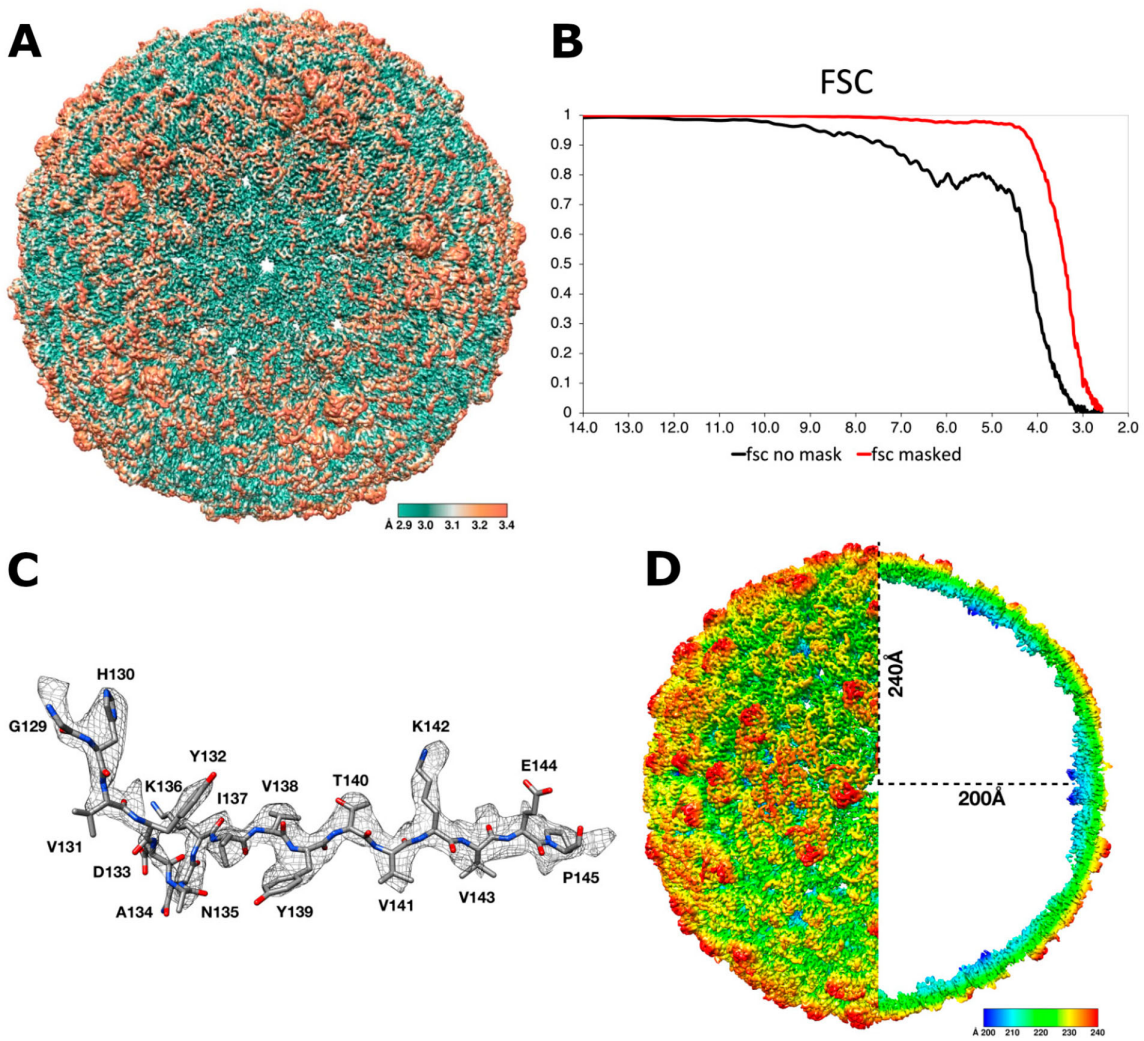
Cryo-EM reconstruction of inactivated TBEV at 3.02 Å resolution. (A) Local resolution map calculated in cryoSPARC with FSC = 0.5; (B) FSC plot; (c) The cryo-EM map around residues 129–145; (D) cross-section of the iTBEV cryo-EM structure coloured by the distance (Å) from the centre of virion.

**Figure 2.**
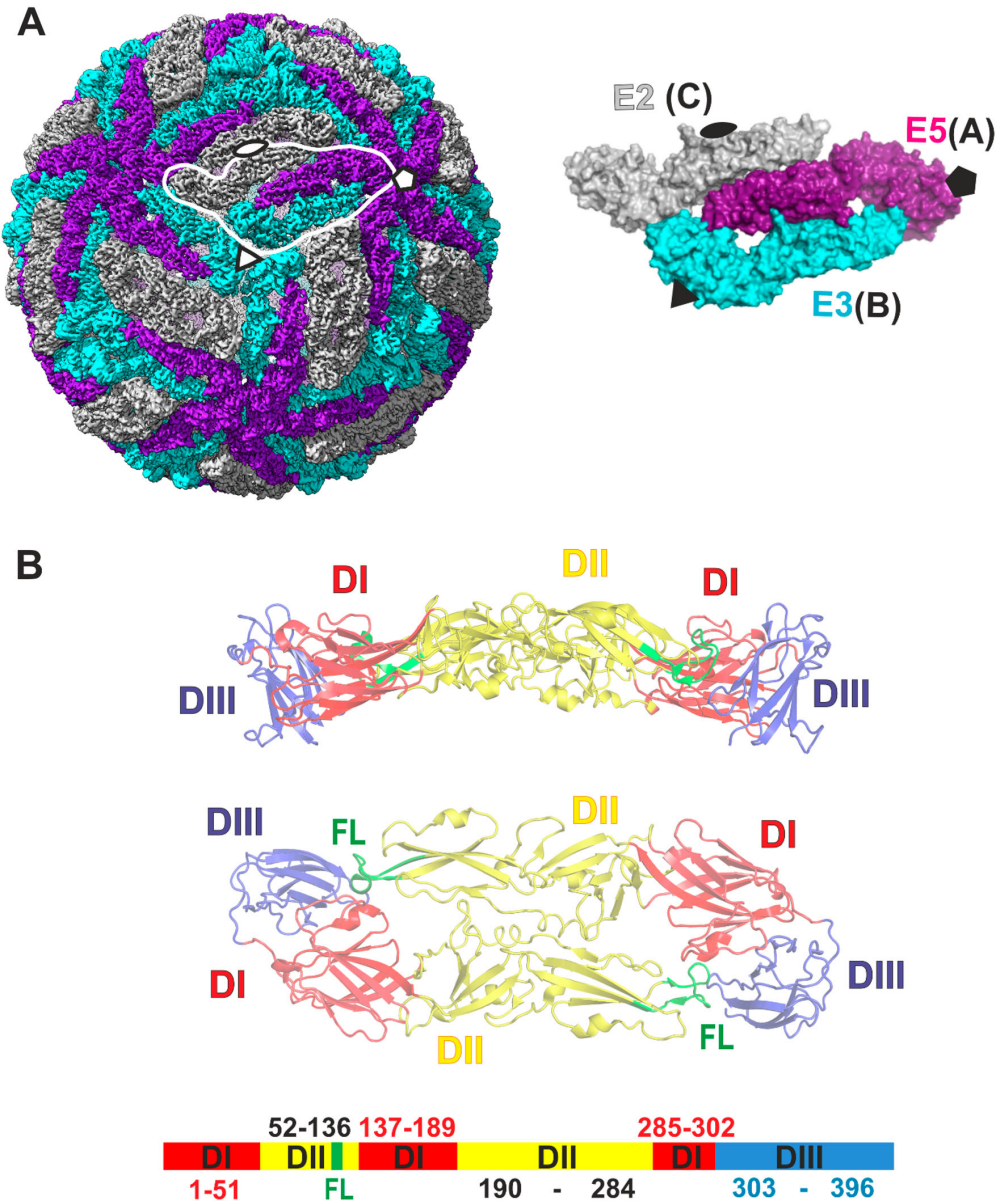
Overall structure of iTBEV. (A) iTBEV E protein ectodomains coloured by symmetrical equivalency. The asymmetric unit and symmetry elements are shown separately. Corresponding subunit IDs in the atomic coordinates file are shown in parentheses. (B) E protein domains, side (upper) and top (below) view. Domain I is coloured red, domain II is coloured yellow and domain III is coloured blue. Fusion loop (residues 98–113) is coloured green. Residue numbering and domain definitions according to [28] are shown as a linear diagram.

The iTBEV cryoEM structure was reconstructed from an electron density map at average resolution of 3.0 Å according to the 0.143 Fourier shell correlation (FSC) criterion (Figure 1, Table S1) [27]. Electron density map illustrated the quality of the structure (Figure 1C). A clear electron density was observed for the E protein ectodomain shell showing the arrangement of antiparallel dimers on the surface of the virion into the common “herringbone” pattern with icosahedral symmetry (residues 1–396, Figure 2). Residues 14–18 (domain I) were not resolved in two of three independent subunits. We also did not observe electron density for any parts of the M protein as well as for stem and anchor regions of E protein (Figure 1D), in contrast to the previously published TBEV structures [23,24]. Disorder in the transmembrane region of our structure is likely caused by the formaldehyde treatment upon the virus inactivation.

Asymmetric unit contained three symmetry-independent E ectodomain subunits, located next to the twofold, threefold, or fivefold icosahedral axes, respectively (referred to as E2, E3, and E5 in this paper by analogy with [29], Figure 2A). Thus two types of dimers are present: symmetric dimer formed by twofold icosahedral axis-related subunits, and asymmetric dimer formed by E3 and E5 subunits (Figure 2).

### E protein subunit interactions

The overall root-mean-square deviation (RMSD) between the С_α_ atoms of E3–E5, E2–E5, and E2–E3 monomers in iTBEV is 0.977, 0.676, and 0.955 Å. It is a bit larger than RMSD for other high-resolution cryoEM orthoflavivirus structures (Supplementary Table S2). Dimer structures are very similar to each other, with RMSD not exceeding 1.4 Å in different variants of alignment (dimer-wise or subunit-wise). Differences as large as 1.6–5.0 Å are observed at several loops of all three monomers and at the C-terminus of iTBEV E5 monomer (Supplementary Table S3).

Оverall folding and structure of E protein ectodomains are very similar to the previously published cryo-EM TBEV structures [23,24] due to high similarity of the sequences. The C_α_ atoms of the refined iTBEV E5, E3, E2 monomers show RMSD of 0.907, 0.921, and 0.977 Å with respect to the UV-inactivated TBEV-Kuutsalo structure and 0.733, 0.842, and 0.817 Å to the intact TBEV-Hypr structure. Buried surface area of ectodomain E dimer interface E5:E3 is 1620.4 Å^2^ for iTBEV, and 1435.8 and 1518.8 Å^2^ for TBEV-Kuutsalo and TBEV-Hypr, respectively.

**Figure 3.**
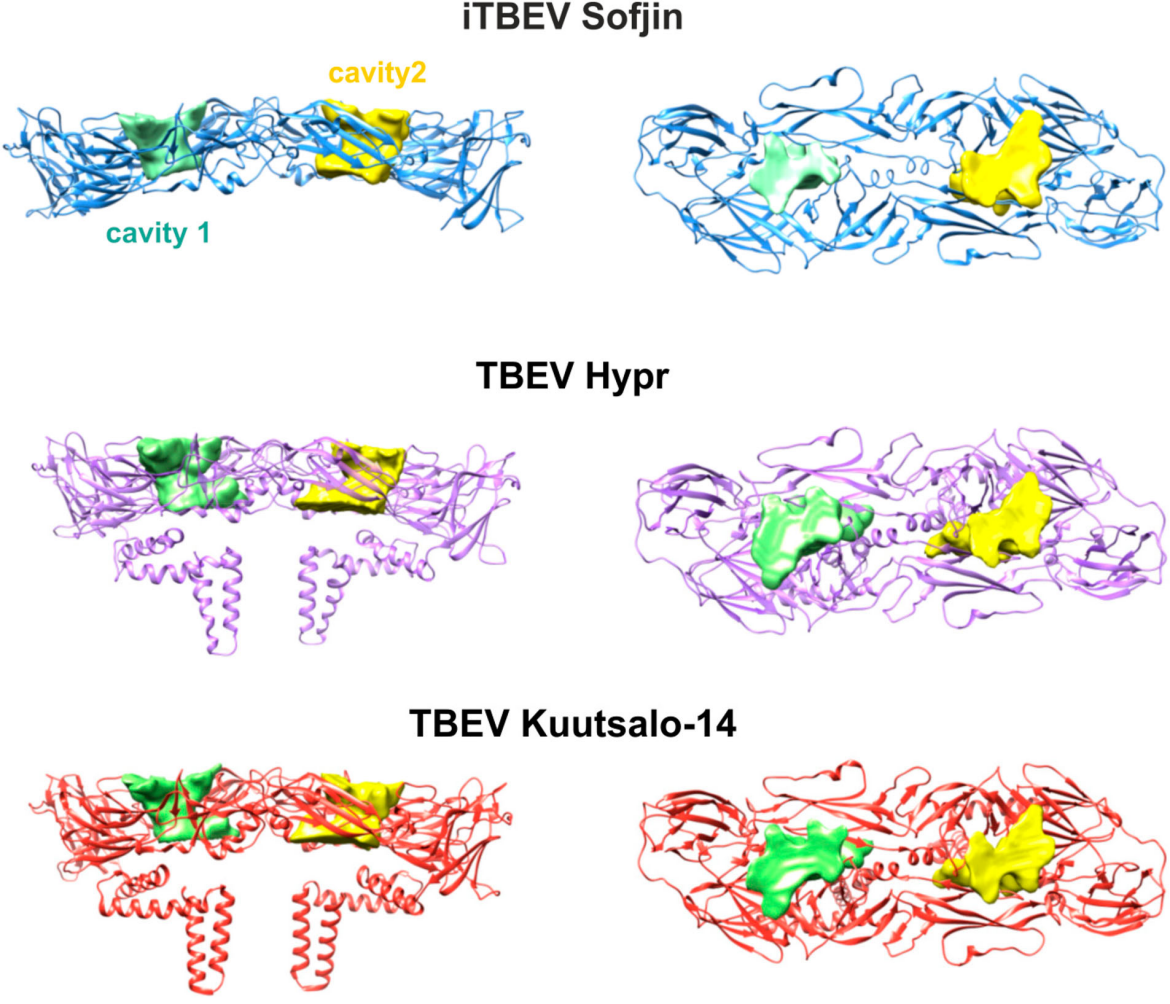
Inter-subunit cavities enclosed by E5–E3 dimers in iTBEV (blue), TBEV-Kuutsalo-14 (red), and TBEV-Hypr (violet). Cavity 1 is shown in green, cavity 2 in yellow. Cavity volume and surface area are given in Supplementary Table S4.

Characteristic cavities in the E protein ectodomain dimer may serve as another measure of dimer asymmetry [29]. These cavities are formed by regions of I and II domains (Supplementary Table S4). We calculated the volume and surface area for these cavities for E5–E3 dimers in all the three TBEV structures (Figure 3), as well as for Zika virus and Japanese encephalitis virus (Supplementary Table S4). iTBEV had the smallest surface area and volume for both cavities compared to those in TBEV-Kuutsalo and TBEV-Hypr, consistent with the largest buried surface area between the subunits. In contrast to the other structures, iTBEV cavities show a noticeable asymmetry: surface area and volume of the cavity 1 are almost 25% smaller than those of the cavity 2.

### Antibody epitope conservation

**Figure 4.**
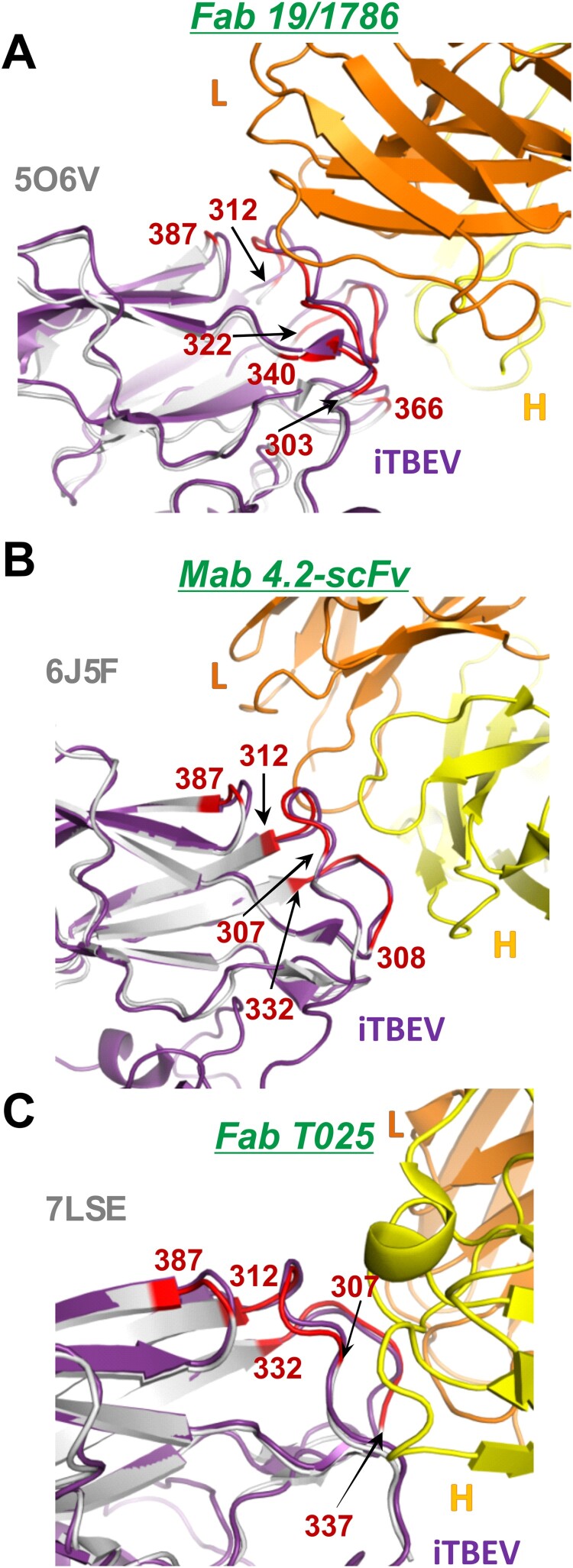
Superposition of the iTBEV E5 subunit with previously published structures of complexes of Fab antibody fragments with E proteins: Fab 19/1786 with TBEV-Hypr (A, PDB ID 5O6V [23]), Mab 4.2-scFv with TBEV-Neudorfl (B, PDB ID 6J5F [32]), and Fab T025 with TBEV-Sofjin (C, PDB ID 7LSE [33]). Fab fragments are coloured orange (light chain) and yellow (heavy chain). iTBEV E5 subunit is coloured violet. TBEV E protein from complex structures is coloured grey.

Protective efficacy of inactivated vaccines depends on the antibody spectrum they elicit. Given the wide applicability and cross-subtype efficiency of TBE vaccines [1,4,5,11,30,31], one may conclude that the inactivation does keep the sufficient epitopes to induce a protective antibody response. High-resolution structure of the inactivated virion provides insight into the conservation of surface epitopes. We have superposed our iTBEV structure to the previously published structures of TBEV complexed with Fab fragment of neutralizing antibodies, a cryo-EM structure of the mature TBEV strain Hypr with Fab 19/1786 (PDB ID 5O6V [23]) and X-ray structures of recombinant E protein domain III from TBEV strain Neudorfl with Mab 4.2 (PDB ID 6J5F [32]) and TBEV strain Sofjin with T025 (PDB ID 7LSE [33]). The superposition of the iTBEV E protein domain III with the corresponding regions of 5O6V, 6J5F, and 7LSE by C_α_ atoms showed that our iTBEV has the same conformation of the main chain for the epitopes of all three antibodies (Figure 4). An X-ray structure of a therapeutic antibody ch14D5 with TBEV E protein DIII was also reported, but not yet published [34]. The superposition of the epitope of this antibody (residues 308–315, 331–336, 367, and 387) on the DIII structure of TBEV-Sofjin (PDB ID 7LSE) with the corresponding regions of our structure also did not reveal substantial differences (Supplementary Figure S2).

### Antigenic properties of native and inactivated TBEV virions

The absence of electron density below the E protein ectodomain shell in our structure of inactivated TBEV suggests that formaldehyde linking likely causes rigidification of the ectodomains, which start to act as a sort of “hull,” while membrane-associated domains become less ordered. The resulting particles still elicit protective antibody response if applied as vaccine antigens. Nevertheless, linkages between the protein subunits were not observed directly in the cryo-EM reconstruction. To confirm that the conformational space explored by TBEV ectodomains is restricted upon inactivation, we performed an ELISA experiment at different pH levels.

**Figure 5.**
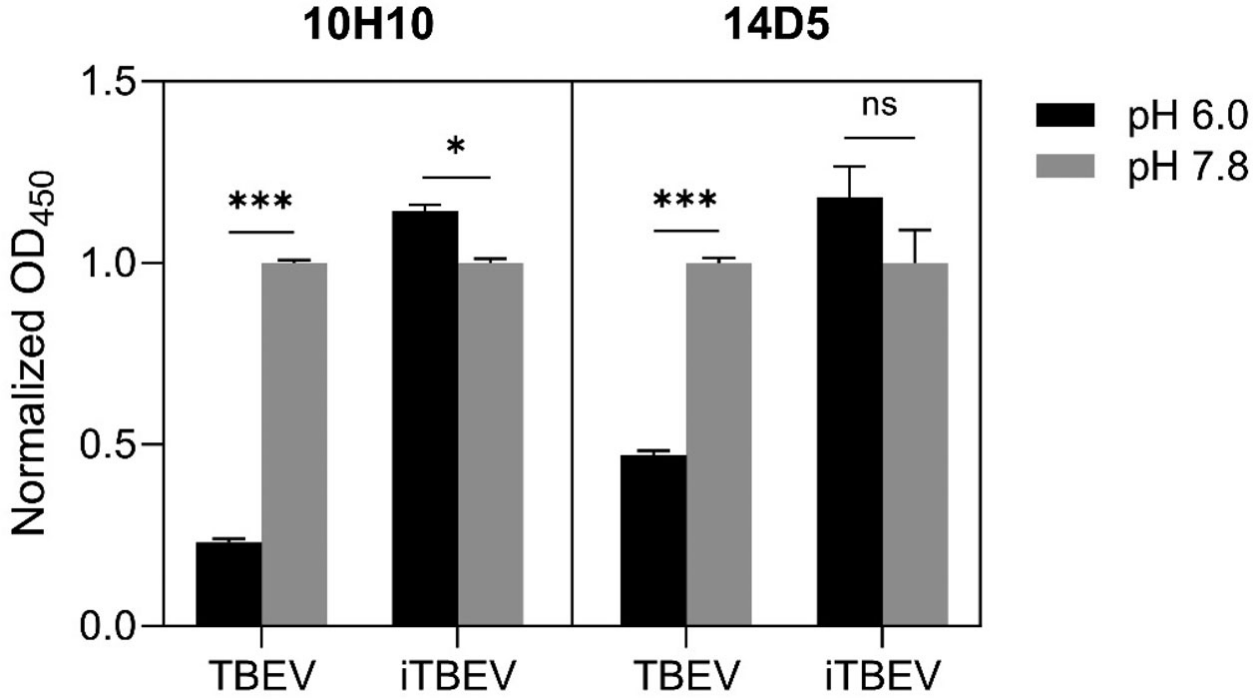
Monoclonal antibody interaction with infectious and inactivated TBEV (ELISA). Decrease of the optical density at 450 nm of TBEV-antibody complexes at pH 6.0 indicates the decrease of antibody binding. Virus samples diluted to the E protein concentration of 10 ng/mL. Optical density normalized to the values at pH 7.8. Statistical significance levels according to the Welch's *t*-test: * *p* ≤ 0.05 and *** *p* ≤ 0.001.

Orthoflaviviruses enter the cells by endocytosis, and the low pH of the endosomes leads to a dramatic conformational rearrangement of the E protein ectodomains and release of the hydrophobic fusion loop on the tip of domain II, which attacks the endosome membrane [35]. Cross-linking of the envelope proteins should prevent the domain rearrangement and the exposure of the fusion loop, and it should be detectable when the inactivated virions are treated with fusion loop antibodies at a lower pH. We have compared the antigenic properties of native and inactivated TBEV upon interaction with cross-reactive DII binding monoclonal antibody (mAB) 10H10 [36] and DIII binding mAB 14D5 [37] at pH 7.8 and 6.0. Optical density at 450 nm decreased substantially with the pH for both mABs bound with the native (infectious) TBEV, while for the inactivated TBEV optical density change was small to negligible (Figure 5, Supplementary Table S5). Therefore, the antigenic structure of the infectious virions changes substantially along with pH, while for the inactivated virions these changes are small, if any, and the virions keep their shape.

## Discussion

Cryo-EM single particle analysis (SPA) [38] is a powerful tool for virus structure determination at an atomic resolution level, being the gold standard for enveloped viruses nowadays. The methodology of Cryo-EM SPA for virology has greatly advanced during past decades [39], allowing to routinely reach resolutions on the level of 3.5 Å. More than 50 cryo-EM structures of orthoflaviviruses or their antibody complexes deposited in the Protein Data Bank (PDB) [40] have resolution better than 11 Å, allowing to visualize bulk structural features of envelope proteins. However, only 15 structures of mature orthoflaviviruses were obtained at a resolution better than 4 Å (Table 1), mostly representing mosquito-borne viruses in the native (non-inactivated) state [29,41–46]. Among the tick-borne orthoflaviviruses, all the currently available high-resolution cryo-EM structures represent the European subtype of TBEV [23–25].

**Table 1.** Cryo-EM structures of mature orthoflaviviruses with the resolution better than 4 Å.

Virus	Strain and serotype	Release year	PDB ID	Resolution, Å	Reference
Dengue virus (DENV)	Thailand/NGS-C/1944 (serotype 2)	2012	3J27	3.6	[41]
			3J2P	3.6	
	Strain 16681 (serotype 2)	2021	6ZQU	3.1	[42]
DENV/BINV	Chimeric flavivirus between Binjari virus (BINV) and DENV serotype 2	2020	7KV8	2.5	[43]
MVEV/BINV	Chimeric flavivirus between BINV and Murray Valley encephalitis virus (MVEV)		7KVB	3.7	
Spondweni virus	SM6 V-1s	2021	6ZQV	2.6	[42]
TBEV	Hypr (European)	2018	5O6A	3.9	[23]
	Kuutsalo-14 (European), UV-inactivated	2022	7Z51	3.3	[24]
	Sofjin-Chumakov (Far-Eastern), formaldehyde-inactivated	2023	8R8L	3.0	This work
			8QRH	3.8	[44]
Usutu virus	SAAR-1776, model A	2021	7LCG	2.42	[45]
	SAAR-1776, model B		7LCH	2.35	
West Nile virus (WNV)	Kunjin	2020	7KVA	3.1	[43]
WNV/BINV	Chimeric flavivirus between BINV and WNV (Kunjin)		7KV9	2.9	
Zika virus	ZIKV/Human/French Polynesia/10087PF/2013	2018	6CO8	3.1	[28]
		2016	5IZ7	3.7	[46]
			5IRE	3.8	[47]

Orthoflaviviruses are highly pathogenic and require biosafety level (BSL) 3 conditions to handle them, limiting the options for structure studies in lower BSL facilities. As a possible solution, insect-specific Binjari virus was suggested as a surrogate for the construction of non-pathogenic chimeric orthoflaviviruses, showing the conservation of the major structural features in the chimeras [43]. The inactivation of the virus is another way to bring the pathogen to the devices installed at low BSL laboratories, as exemplified by the TBEV-Kuutsalo (7Z51) structure representing an UV-inactivated particle, which also retains the features of the structural proteins. Nevertheless, the efficiency of TBEV inactivation by UV irradiation strongly depends on the volume of the sample and its composition even in the μL scale, thus requiring caution in the handling of the treated samples [48]. Chemical inactivation is an alternative approach, which also requires experimental validation for the structural studies; inactivation with formaldehyde is currently the only method used in the TBEV vaccine production. Our 3.0 Å cryo-EM structure of formaldehyde-inactivated TBEV strain Sofjin-Chumakov (iTBEV) from the TBE-Moscow vaccine preparation represents the first structural characterization of a vaccine used in the clinical practice.

Safety and protective efficacy of all the formaldehyde-inactivated TBE vaccines are well established in the *in vitro* and *in vivo* studies and, most importantly, in the clinical practice [1,4,5,11,31]. Thus one may suggest that all the major structural epitopes should be conserved in the vaccine particles and not destroyed by the formaldehyde inactivation. Our structure directly confirms this hypothesis: for all the previously described complexes of TBEV envelope proteins with antibodies, resolved by different methods (X-ray crystallography or cryo-EM), the epitope structure is reproduced by our data, meaning that formaldehyde inactivation is soft enough and suitable for the virus structure studies in general.

On the other hand, inactivation prevents the virus from initiating the infection process, and we also directly confirmed it by the assessment of pH dependence of monoclonal antibody (mAb) binding with intact and inactivated TBEV. While intact TBEV conformationally rearranges at pH 6.0 and decreases its ability to bind mAbs, inactivated TBEV binds them with the same efficiency at pH 6.0 and 7.8, suggesting that its conformation is not dependent on the pH due to formaldehyde-induced cross-linking of the envelope proteins. Although these cross-links are sparse enough to be not observed in the cryo-EM structure due to the stochastic nature of the linking, they are still strong enough to make the “hull” of the E protein ectodomains, which is apparently rather rigid. The rigidity of the hull induces the disorder of the membrane-associated protein regions, making them non-reconstructable. Another putative consequence of the hull cross-linking is the more compact arrangement of protein shell compared to the other TBEV structures.

Our results confirm the general reliability of structural investigations for inactivated virions, which do not require high level biosafety facilities and are thus much more accessible than the studies of infectious virus samples. The influence of chemical inactivation onto the virion structure is rather poorly studied in general, and our data should improve the understanding of this influence based on more thorough comparisons between the structures modified in the different ways. Detailed epitope mapping of the inactivated virion structure and comparative assessment of the vaccine-induced and disease-induced antibody spectra should guide the rational antigen design for improved next-generation vaccines, as well as would serve as a gold standard during the development of vaccines inactivated by other chemical and physical agents.

## Materials and methods

### TBEV sample preparation

iTBEV sample (strain Sofjin-Chumakov [49]) was obtained according to the method developed earlier [26]. Briefly, virus-containing cultural liquid was inactivated with 0.2% formaldehyde, iteratively centrifuged, resuspended, and centrifuged on the sucrose density gradient. Target fractions were identified by ELISA and centrifuged again. The residue was resuspended in the TNE/5 buffer, aliquoted, and kept at −70°C. TBEV concentration was assessed as described [26].

### Cryo-EM data collection and processing

Data processing workflow was described in details earlier [26]. Briefly, we have collected a cryo-EM dataset of inactivated virus sample using an in-house cryo-TEM Titan Krios (Thermo-Fisher Scientific) equipped with the Falcon II detector at 300 kV, 0.863 Å pixel size and a total dose of 64 e^-^/Å/s. Data processing was performed in Warp [50]. Particles extracted with the box of 800 pixels were exported to CryoSPARC [51] and Fourier-cropped to 540 pixels. Multiple rounds of 2D classification were used to remove false-positive picks, followed by heterogeneous refinement. Best class was used for the 3D refinement and CTF refinement. Finally, local refinement with the tight mask covering the virion was performed using 0.05 degrees angular step and the final 3D structure was reconstructed with the Ewald sphere correction resulting in a 3.02 Å resolution. Cryo-EM electron density maps were deposited in the Electron Microscopy Data Bank (https://www.ebi.ac.uk/pdbe/emdb/) under accession number EMD-19003, and the fitted coordinates were deposited in the Protein Data Bank [40] under PDB access code 8R8L.

### Model building and analysis

AlphaFold2 [52] was used to generate an initial atomic model of the E protein ectodomain from TBEV strain Sofjin-Chumakov [49]. The model was fitted into electrostatic potential density using Coot [53], Phenix [54], and Isolde [55]. Finally, two N-glycans covalently attached to Asn154 were added to the model using Coot and refined with additional restrains.

Structure analysis was performed using CCP4 suite [56], PyMol [57], and Coot. Structure-related images were generated using PyMol, Chimera [58], and ChimeraX [59]. The analysis of accessible surface cavities was performed in V3 program [60], which uses the rolling probe method for the calculation of volume. Parameters used: outer probe radius 13.0 Å, inner probe radius 2.0 Å, minimum accessible volume of cavity 1100 Å^3^.

### ELISA with monoclonal antibodies

TBEV antigenicity was assessed against monoclonal antibodies 10H10 [36] and 14D5 [37]. TBEV was obtained according to the same protocol as iTBEV, excluding formaldehyde inactivation step. Antigen concentration in the TBEV and iTBEV samples was performed as described earlier [26]. Aliquots of TBEV and iTBEV (10 μL) were dissolved in 90 μL of TN buffer (0.1 M Tris–HCl, 0.13 M NaCl, рН 7.8) or in 90 μL of 0.23 M phosphate saline buffer (pH 6.0) and incubated for 30 min at 4°C. After incubation, the samples were diluted in carbonate buffer (pH 8.3) to the concentrations of approx. 20 and 10 ng/mL of E protein. Aliquots (100 μL) of these TBEV and iTBEV dilutions were put into the wells of a 96-well plate (Corning, USA). Antigen sorption was performed overnight at 4°C. The plate was washed, 50 μL of mABs 10H10 and 14D5 in 1:5000 and 1:10000 dilutions, respectively, were added to the wells and incubated for 1 h at 37°C. After subsequent washing, 100 μL of 1:5000 anti-mouse IgG rabbit antibodies conjugated with the horseradish peroxidase (Sigma-Aldrich) were added and incubated for 1 h at 37°C. After the incubation, 100 μL of chromogenic substrate TMB (Vector-Best, Russia) was added to the wells. The plate was incubated for 10 min at 25°C, reaction was stopped with 100 μL of stop reagent (Vector-Best, Russia), and optical density was measured at 450 nm (Multiskan Sky Microplate Spectrophotometer; Thermo Fisher Scientific, USA).

## Supplementary Material

Supplement_revised_clean_1
