# Supplementary material for: The structure of inactivated mature tick-borne encephalitis virus at 3.0 Å resolution
Source: Emerg Microbes Infect. 2024 Mar 11;13(1):2313849. doi: 10.1080/22221751.2024.2313849 (PMC10930109; doi:10.1080/22221751.2024.2313849)
Supplement: Supplement_revised_clean_1 [file TEMI_A_2313849_SM1196.pdf]

Supplementary Information

The Structure of Inactivated Mature Tick-Borne Encephalitis Virus at 3.0 Å Resolution

Evgeny B. Pichkur[1], Mikhail F. Vorovitch[2,3], Alla L. Ivanova[2], Elena V. Protopopova[4], Valery B. Loktev[4], Dmitry I. Osolodkin*[2,3], Aydar A. Ishmukametov[2,3], Valeriya R. Samygina*[1]

[1] NRC «Kurchatov Insitute», Moscow, 123182, Russian Federation

[2] FSASI "Chumakov FSC R&D IBP RAS" (Institute of Poliomyelitis), Moscow, 108819, Russian Federation

[3] Institute of Translational Medicine and Biotechnology, Sechenov First Moscow State Medical University, Moscow, 119991, Russian Federation

[4] State Research Center of Virology and Biotechnology "Vector", Novosibirsk, Russian Federation

Corresponding authors: osolodkin_di@chumakovs.su (D.I.O.), lera@crys.ras.ru (V.R.S.)

Table S1. Cryo-EM data collection and model refinement statistics.

Model	**iTBEV**
Cryo-EM data	
Microscope	FEI Titan Krios
Accelerating Voltage, kV	300
Detector	Falcon II
Spherical aberration, mm	<0.1
Magnification	75,000x
Defocus range, µm	-0.6 to -1.8
Micrographs	2,666
Cryo-EM reconstruction	
Particles picked	63,000
Particles refined	39,434
Resolution achieved, Å	3.02
Refinement	
No. of Non-Hydrogen Atoms	
All atoms	9166
Protein residues	1178
Nucleotides	0
Waters	0
Ramachandran Plot	
Favored regions, %	94.78
Allowed regions, %	5.22
Outliers, %	0.0
Deviations from ideal values (RMSD)	
Bond, Å	0.011 (0)
Angle, degrees	1.941 (52)
Rotamer outliers (%)	0.81
Cβ outliers (%)	0.37
CaBLAM outliers (%)	2.42
MolProbity score	1.31
Clash score	1.87
Average B-factor (overall), $Å^2$	87.52

Table S2. RMSD between different monomers in TBEV structures and some other high resolution flavivirus structures (Å).

PDB ID	**E5(A) to E3 (B)**	**E5(A) to E2(C)**	**E3(B) toE2(C)**
**8R8L**	**0.977**	**0.676**	**0.955**
7Z51	0.633	0.467	0.479
5O6A	0.404	0.271	0.236
7KVA	0.530	0.412	0.473
7LCG	0.867	0.549	0.707
7LCH	0.838	0.509	0.679
6ZQV	0.529	0.280	0.451
6ZQU	0.326	0.382	0.420
6CO8	0.77	0.61	0.74

Table S3. Maximum displacement between residues of monomers in asymmetric unit of iTBEV

Monomers	residue	Displacement, Å
**E5 to E3**	16	1.902
	17	2.424
	18	1.837
	19	1.871
	20	1.689
	36	1.985
	37	2.154
	75	2.341
	76	2.084
	77	1.702
	78	2.225
	104	2.263
	105	1.999
	106	2.064
	107	2.102
	170	3.300
	204	5.445
	269	3.034
	270	3.095
	299	2.278
	300	2.382
	301	2.308
	347	2.121
	348	2.948
	349	2.234
	367	2.366
**E5 to E2**	148	1.571
	203	1.912
	204	4.712
	269	2.949
	270	2.542
	348	4.280
	349	2.078
**E3 to E2**	16	3.149
	17	4.705
	18	3.112
	35	1.680
	36	2.441
	37	2.948

Table S4. Surface channels enclosed in ectodomain E-E heterodimer of TBEV, ZIKV, and Japanese encephalitis viruses.

PDB ID	Virus	Resolution, Å	Cavity 1	Cavity2
6CO8	ZIKV	3.1	Volume 2214 A^3 , Surface Area 1411 A^2	Volume 2235 A^3 Surface Area 1477 A^2
			Volume **2038** A^3, Surface Area **1324** A^2 *	
5YWO	JEV	4.3	Volume **3643** Å^3 Surface Area **4288** A^2 *****	
8R8L	iTBEV	3.0	Volume: **2632** Å^3, surface area **1478** Å^2	Volume **3522** Å^3, surface area **1953** Å^2
7Z51	TBEV Kuutsalo-14	3.3	Volume **4037** Å^3, surface area **2134** Å^2	Volume **4282** Å^3, surface area **2229** Å^2
5O6V	TBEV Hypr	3.9	Volume **3846** Å^3, surface area **1994** Å^2	Volume **3750** Å^3, surface area **1989** Å^2

* For E-M pocket enclosed [18], average for two cavities

Table S5. Raw optical density values (two replicates) for interaction of infectious and inactivated TBEV with monoclonal antibodies, normal mouse sera, and anti-mouse IgG at pH 7.8 and 6.0 (ELISA)

Infectious TBEV								
[E], ng/mL	10H10		14D5		Normal mouse sera		Anti-mouse Ig G	
	pH 6.0	pH 7.8	pH 6.0	pH 7.8	pH 6.0	pH 7.8	pH 6.0	pH 7.8
86	1.570<br>1.550	4.296<br>4.486	4.769<br>4.922	4.679<br>4.883	0.361	0.354	0.075	0.081
43	1.180<br>1.197	4.419<br>4.356	3.442<br>3.413	4.979<br>4.855	0.374	0.353	0.069	0.95
21.5	**0.663**<br>**0.648**	**2.910**<br>**2.824**	**1.952**<br>**1.878**	**3.760**<br>**3.620**	0.345	0.346	0.072	0.078
10.8	**0.378**<br>**0.358**	**1.583**<br>**1.601**	**1.106**<br>**1.071**	**2.327**<br>**2.282**	0.338	0.347	0.067	0.077
Inactivated TBEV								
[E], ng/mL	10H10		14D5		Normal mouse sera		Anti-mouse Ig G	
	pH 6.0	pH 7.8	pH 6.0	pH 7.8	pH 6.0	pH 7.8	pH 6.0	pH 7.8
20	**3.731**<br>**3.669**	**3.437**<br>**3.349**	**2.495**<br>**2.476**	**2.464**<br>**2.460**	0.346	0.341	0.067	0.078
10	**2.115**<br>**2.163**	**1.887**<br>**1.856**	**1.308**<br>**1.448**	**1.093**<br>**1.242**	0.351	0.341	0.057	0.068
5	1.130<br>1.112	1.024<br>1.009	0.638<br>0.666	0.634<br>0.643	0.336	0.342	0.055	0.061
2.5	0.639<br>0.687	0.574<br>0.579	0.354<br>0.438	0.325<br>0.355	0.356	0.347	0.057	0.064

Bold values correspond to the linear region and were taken into comparison.

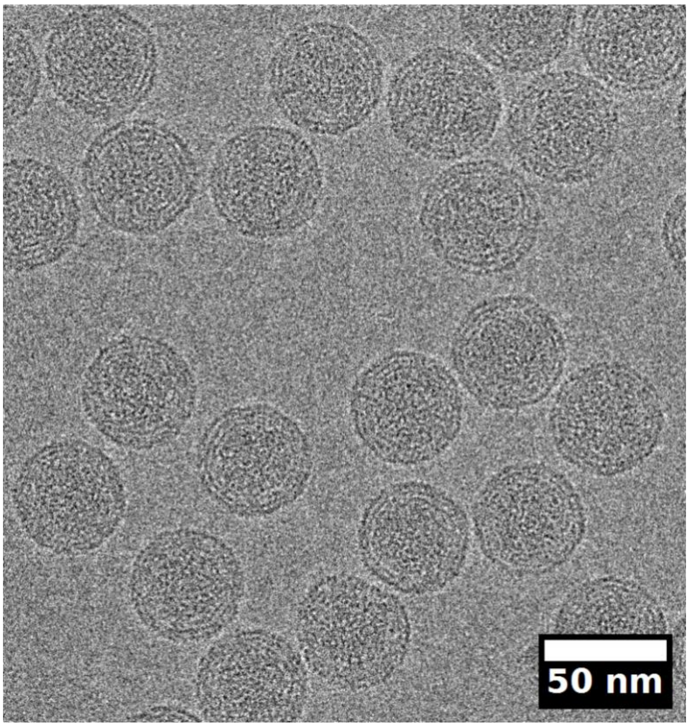

Figure S1. Cryo-EM image of the iTBEV sample demonstrating high purity of the sample and homogeneous particles.

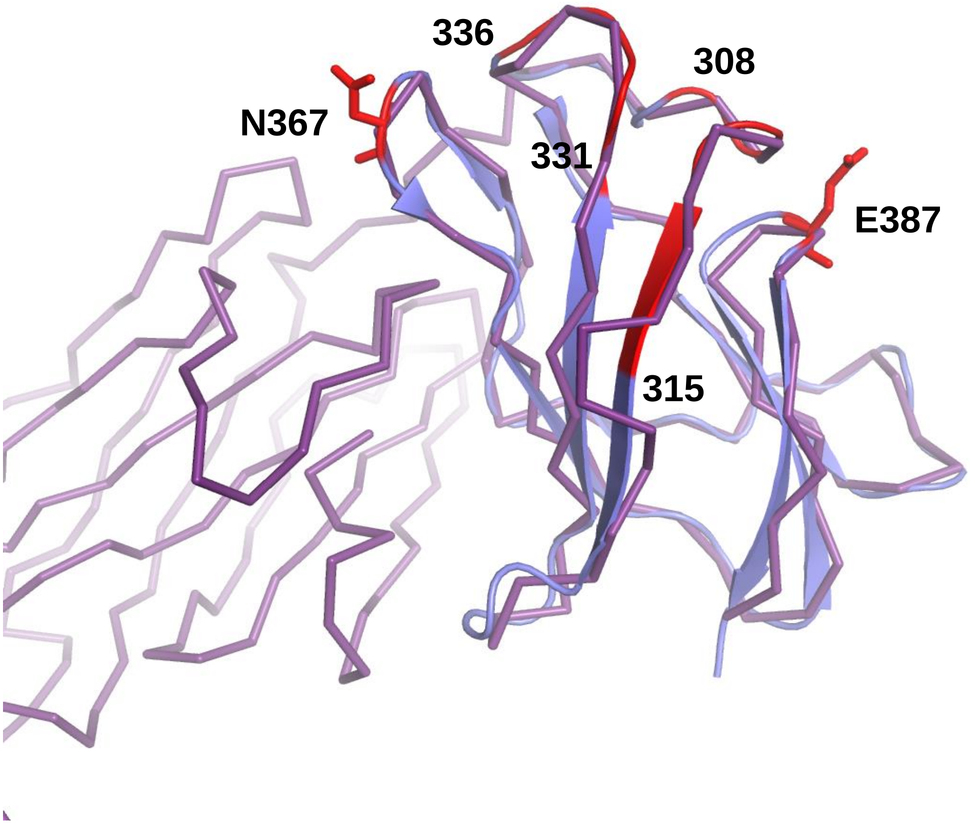

Figure S2. Superposition of iTBEV monomer E5 to DIII of TBEV-Sofjin (PDB ID 7LSE). DIII of 7LSE (blue) is shown in cartoon representation, epitopes for antibody ch14D5 colored red. Residues N367 and E387 are shown in stick representation. Monomer E5 of iTBEV is shown in ribbon representation and colored dark violet.
